# Wound Healing Potential of *Elaeis guineensis* JacqLeaves in an Infected Albino Rat Model

**DOI:** 10.3390/molecules15053186

**Published:** 2010-04-30

**Authors:** Sreenivasan Sasidharan, Rajoo Nilawatyi, Rathinam Xavier, Lachimanan Yoga Latha, Rajoo Amala

**Affiliations:** 1 Institute for Research in Molecular Medicine (INFORMM), Universiti Sains Malaysia, 11800 USM, Penang, Malaysia; 2 Department of Biotechnology, Faculty of Applied Sciences, AIMST University, Jalan Bedong-Semeling, Batu 3½, Bukit Air Nasi, 08100 Bedong, Kedah, Malaysia; E-Mail: xavier@aimst.edu.my (R.X.); 3 School of Biological Sciences, University Sains Malaysia, 11800 USM, Penang, Malaysia; E-Mail: latha_usm@yahoo.com (L.Y.L.); 4 Centre for Drug Research, University Science of Malaysia, 11800 USM, Pulau Pinang, Malaysia; E-Mail: amala_87@yahoo.com (R.A.)

**Keywords:** wound infection, *Elaeis guineensis*, anti-yeast activity, wound healing activity

## Abstract

*Ethnopharmacological relevance: Elaeis guineensis* Jacq (Arecaceae) is one of the plants that are central to the lives of traditional societies in West Africa. It has been reported as a traditional folkloric medicine for a variety of ailments. The plant leaves are also used in some parts of Africa for wound healing, but there are no scientific reports on any wound healing activity of the plant. *Aim of the study:* To investigate the effects of *E. guineensis* leaf on wound healing activity in rats. *Methods:* A phytochemical screening was done to determine the major phytochemicals in the extract. The antimicrobial activity of the extract was examined using the disk diffusion technique and broth dilution method. The wound healing activity of leaves of *E. guineensis* was studied by incorporating the methanolic extract in yellow soft paraffin in concentration of 10% (w/w). Wound healing activity was studied by determining the percentage of wound closure, microbial examination of granulated skin tissue and histological analysis in the control and extract treated groups. *Results:* Phytochemical screening reveals the presence of tannins, alkaloids, steroids, saponins, terpenoids, and flavonoids in the extract. The extract showed significant activity against *Candida albicans* with an MIC value of 6.25 mg/mL. The results show that the *E. guineensis* extract has potent wound healing capacity, as evident from better wound closure, improved tissue regeneration at the wound site, and supporting histopathological parameters pertaining to wound healing. Assessment of granulation tissue every fourth day showed a significant reduction in microbial count. *Conclusions***:** *E. guineensis* accelerated wound healing in rats, thus supporting this traditional use.

## 1. Introduction

Wound infections are most common in developing countries, such as Sub-Saharan African and South Asian countries, than in developed countries. Current estimates indicate that nearly 6 million people suffer from chronic wounds worldwide [1]. The prevalence of chronic wounds in the community was reported as 4.5 per 1000 population, whereas that of acute wounds was nearly double, at 10.5 per 1,000 population [2]. The poor hygienic condition in some third world countries is the main cause of this problem. Besides that, most people in developing countries who suffer from an infected wound cannot afford to purchase modern drugs, which are very expensive and might have side effects. Balick and Cox [3] reported that only 1 to 3% of drugs listed in Western pharmacopoeia are intended for use on the skin and on wounds; in comparison, at least one third of herbal remedies are for such uses. Hence, plant products are seen as alternative solutions to the problem of wound treatment in developing countries. Plant products are potential agents for wound healing, and largely preferred because of their widespread availability and effectiveness as crude preparations. 

The African oil palm (*Elaeis guineensis*) is one of the plants that are central to the lives of traditional societies in West Africa. All parts of this plant are useful. The wood is used as frames for buildings, and the sap is fermented into palm wine. The oil from the fruit mesocarp and the seeds are used for cooking, and making soaps, creams, and other cosmetics. The fresh sap is used as a laxative, and partially fermented palm wine is administered to nursing mothers to improve lactation. The fruit-husk is used in the preparation of soaps used to treat skin infections. A root decoction is used to treat headaches in Nigeria. The pulverized roots are added to drinks as a cure for gonorrhea, menorrhagia, and bronchitis [4]. The leaf extract and juice from young petioles are applied to fresh wounds. The fruit mesocarp oil and palm kernel oil are administered as a poison antidote and used externally with several other herbs as a lotion to treat skin diseases. Palm kernel oil is applied to convulsive children to regulate their body temperature. Oil palm is a folk remedy for cancer, headaches, and rheumatism, and is considered an aphrodisiac, a diuretic, and a liniment [4]. 

Therefore, the economic utilization of leaf material from the oil palm industry waste in Malaysia will be beneficial. Since the oil palm leaf possesses some medicinal value, the current research was conducted to develop a way to convert the agro wastes into an effective wound healing ointment through various biological processes. 

## 2. Results

### 2.1. Phytochemical Analysis

The phytochemical screening of the methanolic extracts of *E. guineensis* is depicted in Table 1. The phytochemical results reveal the presence of tannins, alkaloids, reducing sugars, steroids, saponins, terpenoid, and flavonoids in the extract. 

**Table 1:** Phytochemical Screening of Secondary Metabolites of *Elaies guineensis*.

Secondary metabolites		Methanolic extract
	Tannin	+
	Saponin	+
	Alkaloid	+
	Flavonoid	+
	Steroids	+
	Reducing sugar	+
	Terpenoid	+

Note: + = present, - = absent

### 2.2. Anti-yeast Activity

The results on the fungicidal activity of the extract against *C. albicans* are given in Table 2. The extract exhibited favorable activity against the yeast tested. The zone of clearance produced by the commercial antibiotic disk was larger than that produced by the extract disk. The agar dilution method recorded an MIC value of 6.25 mg/mL.

**Table 2:** Anti-yeast activity (zone of inhibition and MIC^a^) of *Elaies guineensis* extract compared with commercial antibiotic ciprofloxacin.

**Yeast**	**Methanol extract**		
	**Zone of Inhibition (mm)b**		**MIC (mg/mL) of the extract**
*Candida albicans*	**Crude Extract**	**Ciprofloxacin**	
	16.00	21.00	6.25

^a^ Agar dilution method, mean value n = 3; ^b^ The values (average of triplicate) are diameter of zone of inhibition at 10.0 mg/mL crude extract and 5 μg/mL ciprofloxacin.

### 2.3. Microbial Count Analysis

**Figure 1:**
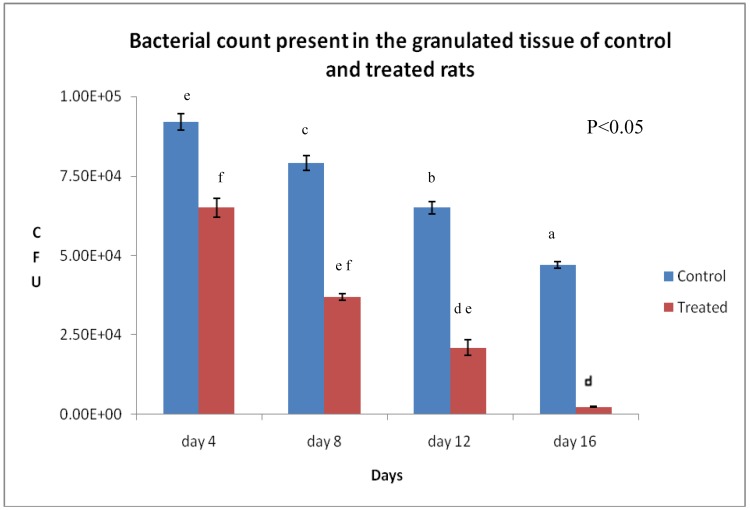
Microbial count present in the granulation tissue of control and infected rats (*n* = 8).

The total bacterial counts from granulation tissue on different days of analysis are shown in Figure 1. The application of plant extract-based ointment resulted in diminishing total bacterial counts in the infected wound. On day 16, the count was decreased to 10^2^ CFU in the treated rats compared to 10^4^ CFU in the control rats. 

### 2.4. Wound Closure Rate

**Figure 2:**
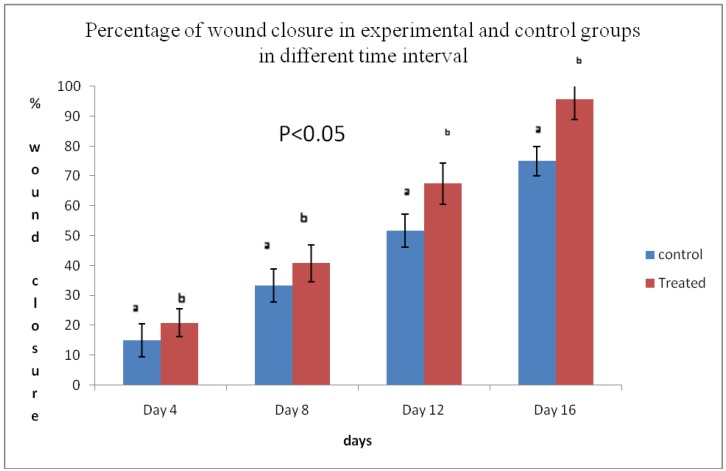
Percentage wound closure in the control and treated groups in different time intervals (*n* = 8). Results are presented as mean ± SD.

**Figure 3:**
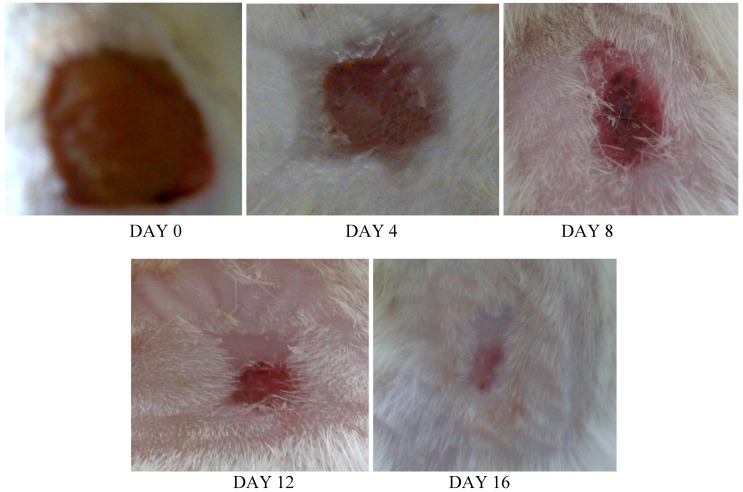
Photographical representation of contraction rate on different days in treatment group.

**Figure 4:**
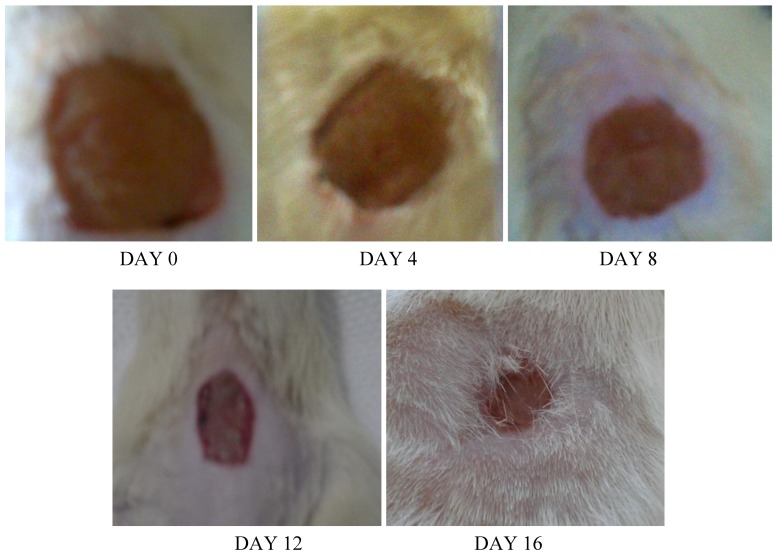
Photographical representation of contraction rate on different days in control group.

A significant difference in wound closure was observed between the two groups from day 4 onwards; in later days, the rate of wound closure in the treated group was much faster than that in the control group. Complete wound closure was observed in the group treated with the *E. guineensis* leaf extract on day 16, whereas it took about 25 days in the control group (Figure 2, Figure 3 and Figure 4).

### 2.5. Histopathology Analysis

**Figure 5:**
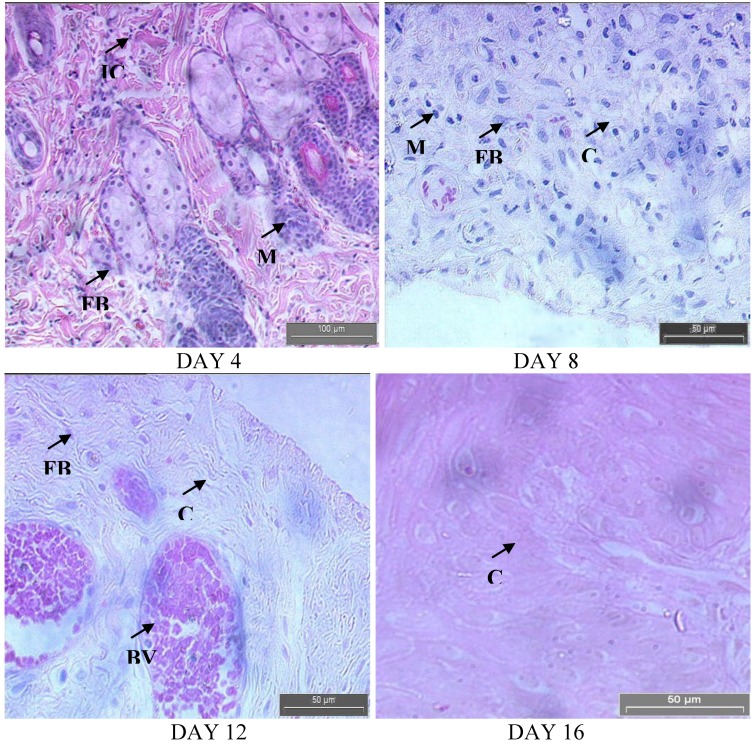
Hematoxylin and eosin stained sections of the granulation tissue in treated group at different time intervals. Fibroblasts (FB), macrophages (M), collagen (C) bundles, Vascularization with larger blood vessels (BV) and inflammatory cells (IC).

**Figure 6:**
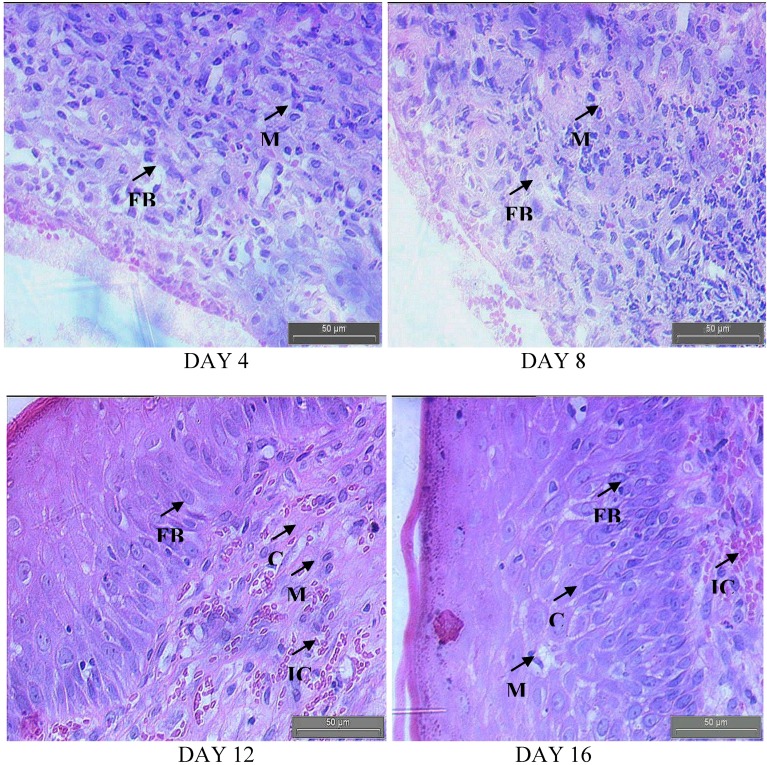
Hematoxylin and eosin stained sections of the granulation tissue in control group at different time intervals. Fibroblasts (FB), macrophages (M), collagen (C) bundles, vascularization with larger blood vessels (BV) and inflammatory cells (IC).

Figure 5 and Figure 6 show the histology of the control and treated rats on different days of analysis, respectively. Complete loss of superficial epithelium and inflammatory exudates were observed in both groups on day 4. The clumps of yeast were higher in the control (Figure 5) compared to the treated rats (Figure 6). 

## 3. Discussion

The present investigation describes some unique features of the leaf extract from the tropical plant *E. guineensis* with respect to its potential wound healing capacity in infected rats. Plant products are potential wound healing agents, and largely preferred because of their widespread availability, non-toxicity, absence of unwanted side effects, and effectiveness as crude preparations. Earlier it was reported that *Centella asciatia* and *Terminalia chebula* are effective in wound healing in rats [5,6]. Various activities were conducted in this study to evaluate the potential of *E. guineensis* as a wound healing agent. One such activity is the phytochemical screening test. The phytochemical results reveal the presence of tannins, alkaloids, reducing sugars, steroids, saponins, terpenoid, and flavonoids in the methanolic extract. The constituents of the oil palm leaf extract, such as terpenoids and alkaloids, may play a major role in the wound healing process observed in this study, however, further phytochemical studies are needed to isolate the active compound(s) responsible for these pharmacological activities [7]. This is because of the presence of terpenoid in the methanolic extract of *E. guineensis*. Terpenoids are known to promote the wound healing process, mainly due to their astringent and antimicrobial properties, which seem to be responsible for wound contraction and an increased rate of epithelialization [8]. Terpenoids, or isoprenoids as they are also known, may have great antifungal or antimicrobial potential due to possible effect on the non-mevalonate pathway. This pathway is essential in fungi, protozoans, gram-negative bacteria and other micro-organisms for the synthesis of cell membrane components, prenylation proteins and as a secondary source of carbon [9]. Studies with other plant materials also demonstrated the presence of similar phytochemical constituents, which were responsible for promoting wound healing activity in rats [10].

The topical application of drugs is an efficient therapy method of destroying microbial populations because the availability of the drug at the infected wound site leads to enhanced wound healing activity. The virulence capacity of microorganisms, amount of inoculums, and host immune response are important factors that can cause massive damage during infection. Normally, common wound pathogens such as *S. aureus*, *C. albicans*, and *P. aeruginosa* with ≥10^3^ CFU/g tissues are classified as infections [11]. From this study, it appears that the *E. guineensis* leaf extract exhibits favorable antimicrobial activity against *C. albicans*. Hence, *C. albicans* infection could be treated with the extract, as the MIC for this extract was found to be only 6.25 mg/mL. Fabry *et al.* [12] reported that if the extracts having activities where MIC values are below 8 mg/mL, this indicates that the extract possesses some effective antimicrobial activity. *In vitro* anti-yeast studies and an *in vivo* short period of epithelialization in the treated rats provide evidence of the healing effect of *E. guineensis* on infected wounds. The bacterial count in the treated rats was significantly reduced to 10² CFU/g tissue on day 16, further confirming the effectiveness of *E. guineensis* treatment, as shown in Figure 1.

*Elaeis guineensis* not only destroys the pathogens from the wound environment; it also acts as a stimulant for wound healing because it has polyphenols and flavonoids as active constituents. After injury, revascularization of the wound bed and redevelopment of the extracellular matrix are achieved through cell proliferation and the production of granulation tissue. Wound contraction, a part of the proliferative phase of wound healing, occurs through the centripetal movement of the tissues surrounding the wound, which is mediated by myofibroblasts [13]. The increased wound contraction in the treated group may be due to the enhanced activity of fibroblasts and successful elimination of yeast by the oil palm leaf extract. The slow rate of wound closure in the control group might be attributed to the presence of microorganisms and their metabolites, which inhibit wound contraction and deteriorates the wound healing activity (Figure 2, Figure 3 and Figure 4). 

A significant increase in collagen content due to enhanced migration of fibroblasts and epithelial cells to the wound site was observed during the wound healing process in the treated group. Moreover, as shown in previous studies, oil palm contains ascorbic acid, which acts as a cofactor for the synthesis of collagen as well as elastin fibers [14]. The decreased collagen content in the control group might be due to a prolonged inflammatory phase where the degradation of collagen will be greater than its synthesis. 

A close examination of granulation tissue sections revealed that tissue regeneration was much quicker in the treated group compared to that in control wounds (Figure 5 and Figure 6). The increased cellular infiltration observed from hematoxylin and eosin staining in both groups may be due to the presence of pathogens, but the antimicrobial property of *E. guineensis* massively reduced the bacterial population, thereby indirectly reducing the inflammatory cells on the wound site. Early dermal and epidermal regeneration in the treated group confirmed that the ointment containing the *E. guineensis* extract had a positive effect toward cellular proliferation, granulation tissue formation, and epithelialization. The well-formed collagen bundles in the treated group shown in hematoxylin and eosin staining support the efficacy of *E. guineensis* on fibroblast proliferation and synthesis of extracellular matrix during healing (Figure 5). Incomplete epithelialization with less extracellular matrix synthesis was observed in control rats, as shown in Figure 6. Clumps of degenerating neutrophils, necrotic changes, and the persistence of inflammatory exudates in the upper dermis with loss of epidermis were also observed up to day 16. The treated rats showed marked epithelialization, a moderate amount of extracellular matrix synthesis, and new blood vessel formation.

## 4. Experimental

### 4.1. Sample Collection

Fresh leaves of *E. guineensis* were collected from various areas in Peninsular Malaysia in July 2008. The leaves were separated and cut into small pieces, which were first washed with tap water and then with distilled water. The leaves were then dried in an oven at 60 ^ο^C for 7 days, after which the dried leaves were ground into fine powder using a grinder.

### 4.2. Extraction Procedure

Dried sample (approximately 100 g) was added to methanol (300 mL) and soaked for 4 days at room temperature (30 ± 2 °C). The suspension was stirred from time to time to allow the leaf powder to fully dissolve in the methanol. Removal of the sample from the solvents was done by filtration through cheesecloth followed by filter paper (Whatman No. 1); the filtrate was concentrated under vacuum to one-fifth its volume using a rotary evaporator at 60 °C and then sterilized by filtration using a 0.22-mm membrane for antimicrobial assay [15]. The thick paste obtained was further dried in an oven at 40 °C. The resultant extract was kept at 4 °C for further analysis

### 4.3. Phytochemical Screening

#### 4.3.1. Saponins

The extract (300 mg) was boiled in 5 mL water for 2 minutes. Then the mixture was cooled and mixed vigorously, and left to stand for 3 min. The formation of froth indicates the presence of saponins [16].

#### 4.3.2. Tannins

To 1 mL of extract (300 mg∙mL^−1^) was added to 2 mL of sodium chloride (2%), filtered and mixed with 5 mL 1% gelatin solution. Precipitation indicates the presence of tannins [16].

#### 4.3.3. Terpenoids

The extract (300 mg) was mixed with 5 mL chloroform and warmed at 80 °C for 30 min. A few drops of concentrated sulfuric acid was added and mixed well into the mixture. The appearance of a red color indicates the presence of terpenoids [17].

#### 4.3.4. Alkaloids

The extract (300 mg) was digested with 2M HCl, and the acidic filtrate was mixed with amyl alcohol at room temperature. The pink color of the alcoholic layer indicates the presence of alkaloids [18].

#### 4.3.5. Phenolics

Diluted NaOH, followed by diluted HCl, was added to the methanolic extract of the sample residue. The solubility and color change of the mixture were noted. A yellow solution with NaOH, which turns colorless with the addition of diluted HCl, confirms the presence of flavonoids [17]. 

#### 4.3.6. Benedict’s Test

Two mg of the methanolic extract was re-dissolved in 2 mL water on the water bath of this solution; 1 mL of Benedict’s solution was added into the test tube. The mixture was shaken and heated in a water bath for 10 minutes. Then its color was recorded. A brick red precipitate indicates the presence of reducing sugars [17].

### 4.4. Anti-yeast Activity

#### 4.4.1. Microorganism

*Candida albicans* was obtained from a laboratory stock culture and used as the test organism. The yeast was cultured on Sabouraud dextrose agar at 30 °C for 24 h. The stock culture was maintained on Sabouraud dextrose agar slants at 4 °C.

#### 4.4.2. Fungicidal activity

The fungicidal activity of the extract was determined following the method described by the NCCLS [19], with slight modifications.

#### 4.4.3. Disk diffusion technique

The test microbe was removed aseptically with an inoculating loop and transferred to a test tube containing 5 mL sterile distilled water. Sufficient inoculums were added until the turbidity was equal to 0.5 McFarland (10^8^ colony-forming units mL^−1^) standard (bioMerieux, Marcy Petoile, France). One milliliter of the test tube suspension was added to 15–20 mL of Sabouraud dextrose agar before the seeded agar plate (9 cm in diameter) was left to solidify for 15 min. Nine Whatman filter paper No. 1 disks of 6 mm diameter were used to screen the fungicidal activity. Each sterile disk was impregnated with 20 μL of extract (corresponding to 100 mg/mL of crude extract), ciprofloxacin (5 μg/mL) (as positive control), and 10% DMSO (v/v) (as negative control). The disks were placed on the surface of the seeded plates, incubated at 37 °C overnight, and examined for zones of growth inhibition.

#### 4.4.4. Determination of minimum inhibitory concentration (MIC)

A 16-h culture was diluted with a sterile physiologic saline solution (PS; 0.85% (w/v) sodium chloride) with reference to the 0.5 McFarland standard to achieve inoculums of approximately 10^6^CFU mL^−1^. A serial dilution was carried out to give final concentrations of between 1.563 and 200.00 mg crude extract per milliliter. The tubes were inoculated with 20 μL yeast suspensions per milliliter nutrient broth, homogenized, and incubated at 37 °C. After incubation, 50 μL was withdrawn from each tube, inoculated on agar plates, and incubated at 37 °C for 24 h. The MIC value was determined as the lowest concentration of crude extract in the broth medium that inhibited the visible growth of the test microorganism.

### 4.5. Wound Healing Activity

#### 4.5.1. Crude extract formulation

A 10% (w/w) crude extract of the oil palm leaf was prepared by mixing the extract (5 g) in yellow soft paraffin (50 g) obtained from a pharmacy [20].

#### 4.5.2. Animal

Sixteen rats weighing between 150 and 200 g obtained from the AIMST University animal house were used. The rats were placed in a room with controlled cycles of 12 h of light and 12 h of darkness; the light went on at 7:00 am. Water and food were provided to the animals *ad libitum*. All the rats were divided into two groups, namely, the treatment group and the control group. Experiments were conducted in accordance with the internationally accepted principles of laboratory animal use and care (EEC Directive of 1986; 86/609/EEC) and the AIMST University Animal Use Guidelines.

#### 4.5.3. *In vivo* Wound Healing Activity

The rats were anaesthetized using 45 mg/kg of diethyl ether given by the intraperitoneal route. A full-thickness wound (1.5 × 1.5 cm) was made on a shaved dorsal area and inoculated with 0.1 mL *Candida albicans* (approximately 10^9^ CFU). After 24 h, the wounds were treated topically with 10% formulated crude extract while the control rats were treated only with yellow soft paraffin for 16 days. The decrease in wound diameters during the healing process was measured with an analytical perimeter. 

#### 4.5.4. Microbial Examination of Granulated Skin Tissue

The granulated tissues from both treatment and control groups were excised prior to application of ointment formulation on day 4, 8, 12, and 16 using sterile scissors and forceps. One mg of excised tissue was placed in 10 mL of sterile saline and vortexed for few minutes. The total cell count was analyzed by serial dilution method.

#### 4.5.5. Histological Analysis

Skin tissues were collected and transferred to 10% neutral buffered formalin (NBF) for 24 h at 4 °C. The formalin-fixed tissues were dehydrated through grades of alcohol and cleared in xylene, and then embedded in paraffin wax (58–60 °C m.p.). Five to 7 μm sections were deparaffinized and stained with hematoxylin, and then counterstained with eosine [21].

### 4.6. Statistical Analysis

All results were expressed as mean ± S.D., and the results were compared statistically by one-way ANOVA using SPSS software (Student Version 14.00). A *P* value < 0.05 was considered statistically significant.

## 5. Conclusions

The above data suggest that the application of *E. guineensis* ointment to an infected wound not only reduces the risk of further infection, but also improves the healing activity. The application of a methanolic extract of *E. guineensis* was found to improve the different phases of wound repair, including collagen synthesis and maturation, wound contraction, and epithelialization. As *E. guineensis* possesses an antifungal property and is traditionally used in several African countries, our findings may provide scientific rationale for the use of *E. guineensis* to promote healing of infected wounds.
